# Corneal endothelium features in Fuchs’ Endothelial Corneal Dystrophy: A preliminary 3D anterior segment optical coherence tomography study

**DOI:** 10.1371/journal.pone.0207891

**Published:** 2018-11-29

**Authors:** Claudio Iovino, Maurizio Fossarello, Giuseppe Giannaccare, Marco Pellegrini, Mirco Braghiroli, Giuseppe Demarinis, Pietro Emanuele Napoli

**Affiliations:** 1 Department of Surgical Sciences, Eye Clinic, University of Cagliari, Cagliari, Italy; 2 Clinica Oculistica, San Giovanni di Dio Hospital, Azienda Ospedaliera Universitaria di Cagliari, Cagliari, Italy; 3 Ophthalmology Unit, S. Orsola-Malpighi University Hospital, University of Bologna, Bologna, Italy; Weill Cornell Medicine-Qatar, QATAR

## Abstract

**Purpose:**

To evaluate the feasibility of 3D anterior segment optical coherence tomography (AS-OCT) for the detection of corneal endothelial features in patients with Fuchs’ Endothelial Corneal Dystrophy (FECD).

**Methods:**

Twenty patients with clinical diagnosis of FECD (group A), and 20 control subjects (group B) were enrolled. In all patients a complete ophthalmological examination was performed, including best corrected visual acuity (BCVA), slit lamp examination for subjective grading of FECD and corneal endothelial specular microscopy. A 512x128 AS-OCT cube centered on the corneal apex was performed, and then the inner surface of the cornea was visualized and analyzed individually.

**Results:**

Overall, the study participants were adults (mean age was 57.35 ± 8.45 years [mean ± SD] 80% female) with a BCVA ranged from 1.3 to 0 LogMAR. The OCT analysis disclosed three different patterns of the corneal endothelium (1, 2, 3) according to the signal distribution and the level of reflectivity: a homogenous, hypo-reflective surface (pattern 1); the presence of hyper-reflective orange-yellowish points (pattern 2); and a mottled appearance with a variable number of hyper-reflective areas (pattern 3). The distributions of these morphological models in the two populations were as follows: patterns 1, 2 and 3 were observed respectively in 0%, 80%, and 20% of patients in group A, and in 80%, 20% and 0% of subjects in group B. Correlation analysis unveiled a positive relationship between OCT corneal endothelium reflectivity and the clinical severity score (assessed with biomicroscopy), as well as an inverse relationship between the OCT pattern and the integrity of corneal endothelium.

**Conclusion:**

3D AS-OCT is a useful tool in investigation of endothelial features and therefore may represent a valuable support in the setting of FECD diagnosis and staging.

## Introduction

Fuchs’ endothelial corneal dystrophy (FECD) is a bilateral and slowly progressive corneal disease, first described in 1910 by Austrian ophthalmologist Ernst Fuchs [1]. It affects approximately 5% of individuals over 40 years of age, and has a predilection for women at a ratio of 2.5–3.1 [2,3]. Although the FECD etiology is not yet fully understood, genetic alterations are considered the major risk factor [4], and several genetic loci have been identified [5].

Despite FECD generally becomes clinically manifest in the fifth or sixth decade of life, an early- and late-onset variant have been reported [6,7]. The latter form, which is the most common corneal endothelium disorder, has an autosomal dominant inheritance with variable penetrance, whereas early-onset disease has an autosomal dominant with a Mendelian inheritance pattern [8,9].

The first clinical manifestation of the disease is the presence of corneal guttae, deposition of focal excrescences that can be observed by a slit-lamp examination generally before the development of any subjective symptoms. The natural history of the disease implies a decrease of the Na-K ATPase pumps in endothelial cells over the time, thus resulting in stromal edema and/or full thickness edematous opacity with visual acuity impairment due to loss of corneal transparency. In the late stages of the disease painful epithelial bulla can also develop [10].

Since FECD represents the most common reason for corneal transplantation among all corneal dystrophies in many countries [11], a prompt and correct diagnosis is mandatory for Ophthalmologists. FECD can be diagnosed by the presence of the following findings: guttae or more advanced corneal alterations by slit lamp examination, the presence of hypo-reflective areas in endothelial background by confocal biomicroscopy, decreased endothelial cell counts by specular microscopy, and an increased corneal thickness measured by pachymetry. More recently, Siebelmann et al analyzed the role of anterior segment optical coherence tomography (AS-OCT) in detecting FECD-related corneal alterations [12], but, to the best of our knowledge, no one paper reports the role of 3D AS-OCT.

The aim of the present study was to analyze a variable number of morphologic features and quantitative parameters of the inner corneal surface in patients with FECD and in healthy subjects by means of 3D AS-OCT. In addition, we evaluated a potential relationship between OCT findings and clinical or instrumental parameters.

## Materials and methods

### Subject and examination

Twenty consecutive FECD patients referring to our clinic for a control visit from January 2017 to December 2017 were included in the study (Group A). Twenty healthy age and gender-matched subjects acted as controls (Group B). The right eye of each participant was considered as the study eye. All investigations and examinations were performed in accordance with the tenets of the Declaration of Helsinki 2013. The study was approved by the local Office of Research Ethics of the University of Cagliari (Italy). After receiving a detailed explanation of the study, written informed consent was obtained from all subjects before examination.

Patients underwent a comprehensive ophthalmologic evaluation encompassing: clinical history, best corrected visual acuity (BCVA), slit lamp examination, endothelial specular microscopy, and AS-OCT imaging (with the cross-line and *3D* scanning modalities in order to quantify the central corneal thickness and to evaluate any corneal endothelial alteration, respectively).

Subjects were excluded from the study if they were affected from the following conditions (which may influence the corneal structure/thickness and the FECD grade):

1) any ocular surface diseases, investigated as previously described [13–15];
2) previous corneal or intraocular surgery;
3) history or any kind of ocular trauma;
4) evidence of corneal dystrophy different from FECD;
5) history of glaucoma or long term ocular medications.

All clinical evaluations were performed during the same period of the day (3 pm to 6 pm).

### Visual acuity

The BCVA was measured with Early Treatment Diabetic Retinopathy Study charts, and analyzed by logarithm of the minimum angle of resolution (logMAR) for statistical analysis.

### Slit lamp biomicroscopy

Grading score of FECD was established by evaluating the cornea both horizontally and vertically from the center to the limbus by slit lamp biomicroscopy using a narrow slit beam [16], staging each eye as suggested in a previous classification proposed by Repp et al [17].

Corneas with a number of central or paracentral, non-confluent guttae (i.e. grade 1 and 2) were classified as affected by mild FECD; corneas with confluent guttae of 1–2 mm and 2–5 mm in size (i.e. grade 3 and 4) were considered as affected by moderate FECD; corneas with confluent guttae >5 mm in diameter with or without any stromal or epithelial edema (i.e. grade 5 and 6) were staged as affected by severe FECD.

Two corneal specialists (MF, PEN) examined all study participants performing a biomicroscopic examination to determine the presence and/or the extent of corneal guttae, as well as the presence of any stromal or epithelial edema. Any disagreement between the two corneal specialists was resolved by a third examiner (CI). Then, a grade was assigned to each affected eye.

### Specular endothelial microscopy

Endothelial specular microscopy (CEM-530; Nidek, Hiroishi, Japan) was performed in all study eyes in order to analyze the following parameters: mean endothelial cells count (CC), coefficient of variation (CV), endothelial cell density (ECD), percentage of hexagonal cell (Hex).

### Spectral-domain OCT

All images were acquired by using a commercially available OCT device, Cirrus HD-OCT 5000 (Carl Zeiss Meditec Inc, Dublin, California, USA). This is a spectral-domain OCT with a 5 μm axial resolution, a wavelength of 840nm and takes 68,000 axial scans per seconds. One drop of artificial tear was instilled 5 minutes before the OCT examination to avoid any artifact from the ocular surface.

The Anterior Segment Cube 512x128 scanning protocol (which acquires a series of 128 horizontal scan lines, each composed of 512 A-scan creating a 3D image of the data) was used for the pattern analysis. In this case, the anterior surface of the cube (3D image) was representative of the outer corneal layers (e.g. the ocular surface) and therefore was excluded from our examination; whereas turning upside down the cube by the embedded software, the innermost layer of the cube was representative of the corneal endothelium alone or in combination with the Descemet’s membrane (DM).

Conversely, the Anterior Segment 5 Line Raster scanning protocol was used to in-depth evaluate the corneal structure and to measure the central corneal thickness.

All OCT scans were analyzed by Image J 1.50 software (National Institutes of Health, Bethesda, MD) to investigate the qualitative and quantitative features of the inner surface of the cornea. Each single corneal endothelium image was divided into 4 quadrants of the same size (supero-temporal, ST; supero-nasal, SN; infero-temporal, IT; infero-nasal, IN), and the mean and maximum reflectivity was calculated for each region.

All 3D AS-OCT images were classified in three different patterns according the signal distribution and the level of reflectivity (low, moderate, and high). We defined the apparent reflectivity based on the following grading scale: from black to blue was absence or low reflectivity, from green to yellow was moderate reflectivity, and red was high/intense reflectivity.

**Fig 1:**
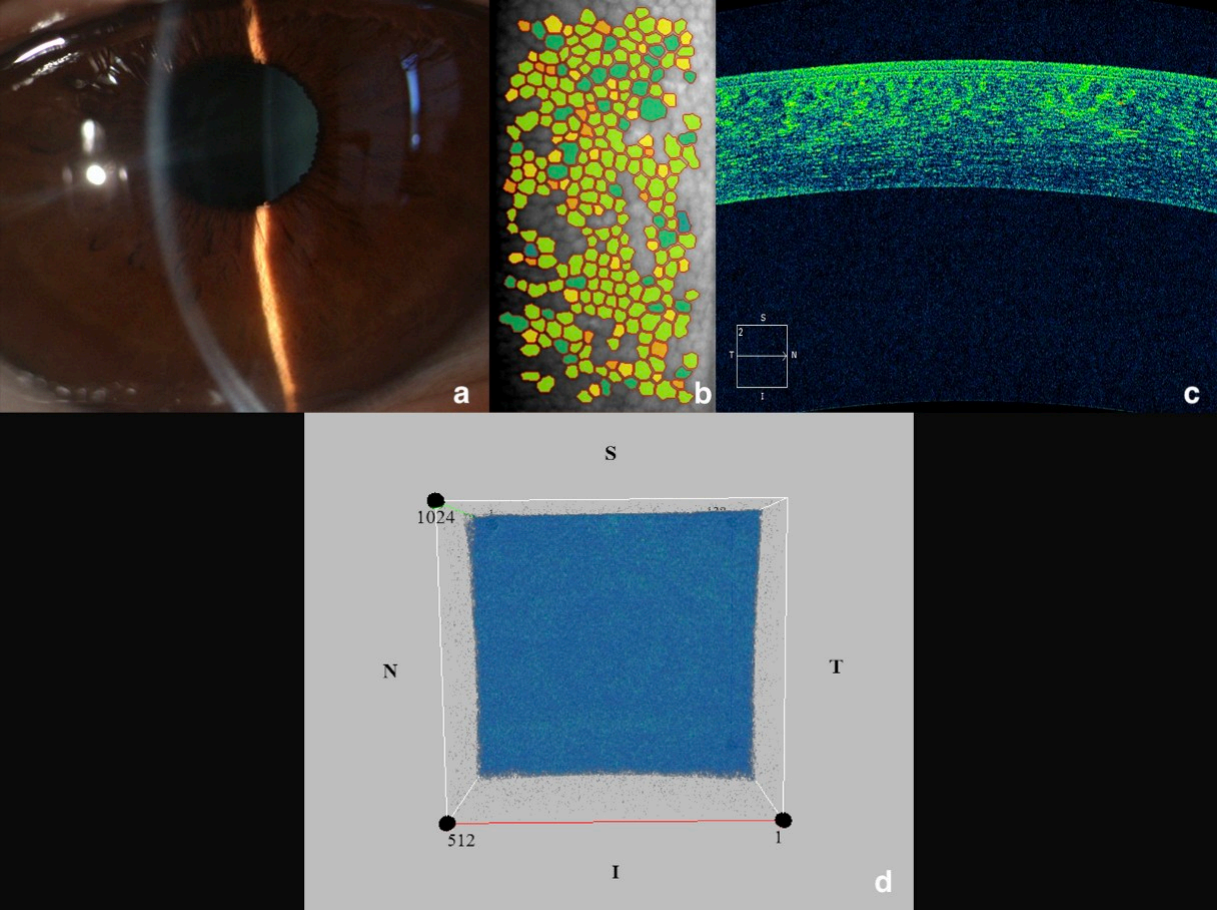
The study eye of a healthy subject and its related OCT pattern n. 1 (‘homogeneous’ appearance). (a) slit-lamp biomicroscopy; (b) specular endothelial microscopy; (c) cross-sectional AS-OCT image confirming a normal corneal structure; (d) 3D AS-OCT image of a 512x128 cube scan showing a homogenous reflectivity of the inner corneal surface, with no hyper-reflective points/areas.

**Fig 2:**
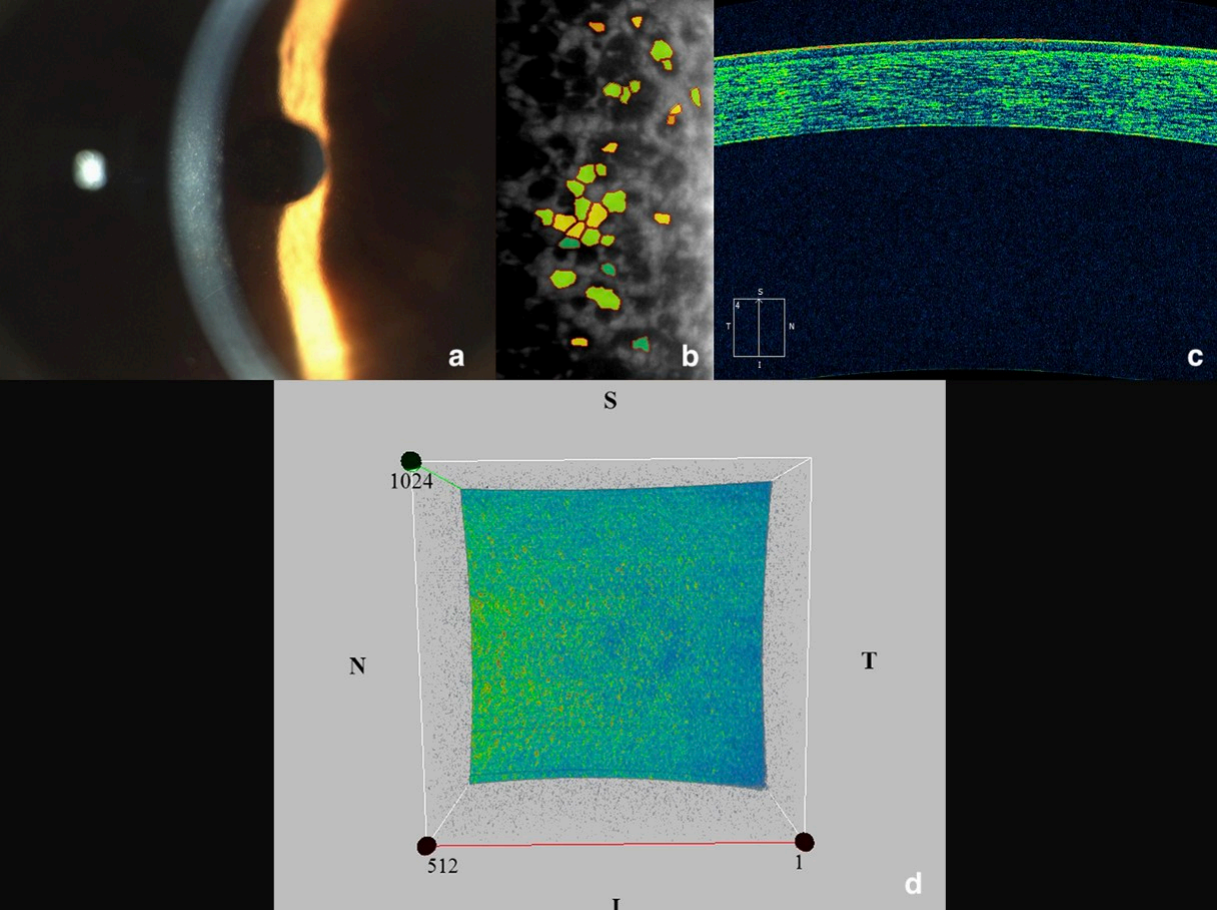
The study eye of a patient with moderate FECD and its related OCT pattern n. 2 (‘dotty’ appearance). (a) slit-lamp biomicroscopy showing a beaten metal-like appearance of the corneal endothelium with the characteristic confluent guttae; (b) specular endothelial microscopy confirming the loss of endothelial cells; (c) cross-sectional AS-OCT scan displaying a faint hyper-reflectivity of the corneal endothelium; (d) 3D AS-OCT image of a 512x128 cube scan showing a large number of hyper-reflective orange-yellowish points scattered through the four quadrants of the inner corneal surface.

**Fig 3:**
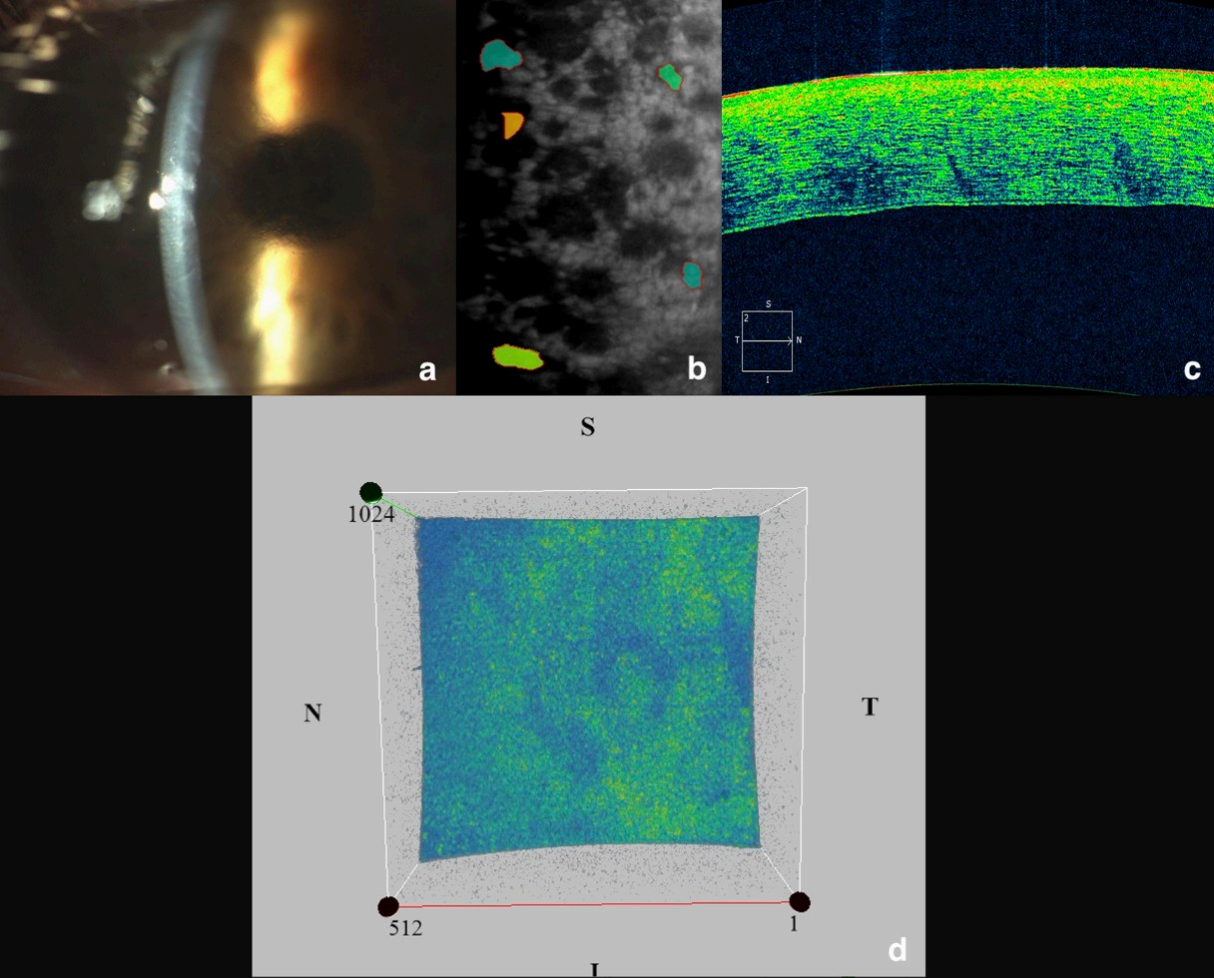
The study eye of a patient with severe FECD and its related OCT pattern n. 3 (‘marmoreal’ appearance). (a) slit-lamp biomicroscopy showing important alterations of the cornea including the presence of endothelial guttae and full-thickness edematous opacities; (b) specular endothelial microscopy confirming the advanced stage of the disease with few residual endothelial cells; (c) cross-sectional AS-OCT scan disclosing irregularities of all corneal layers with a non-homogeneous reflectivity; (d) 3D AS-OCT image of a 512x128 cube scan showing a mottled appearance of the inner corneal surface with a variable number of hyper-reflective areas, resembling a marmoreal look-like.

Consequently, pattern 1 was characterized by a homogenous, and low reflectivity (Fig 1); pattern 2 was defined by the presence of hyper-reflective orange-yellowish points scattered through the four quadrants (moderate reflectivity) (Fig 2); and pattern 3 was characterized by a mottled appearance of the entire inner surface with a variable number of hyper-reflective areas, resembling a marmoreal look-like (moderate/high reflectivity) (Fig 3). For simplicity, pattern 1 was defined as ‘homogeneous’, pattern 2 as “dotty”, and pattern 3 as “marmoreal”.

As described above regarding the biomicroscopic classification, two corneal specialists (MF, PEN) scored the OCT images. Any disagreement between the two corneal specialists was resolved by a third examiner (CI).

### Statistical analysis

Data analysis was performed via computer (SPSS version 21.0). Parametric and nominal data were summarized as mean ± standard deviation (SD), and as percentage, respectively. Normality of data was tested by Shapiro-Wilk test and Lilliefors test. If data demonstrated a normal distribution, then parametric statistical tests were used, otherwise nonparametric tests were used.

Correlation analysis between variables was performed calculating Spearman’s ρ test and Kendall’s τ test in all participants, and for each group separately.

Accepting an alpha risk of 0.05 and a beta risk of 0.2 in a two-sided test, a sample size of 38 is necessary to demonstrate a statistical significance with a correlation coefficient of 0.445. It has been anticipated a drop-out rate of 0%.

The p values less than 0.05 were considered significant.

## Results

Overall, we studied 40 eyes of 40 subjects (57.35 ± 8.451 years [mean ± SD], 80% female). Descriptive statistics for diagnostic tests are provided in Tables 1 and 2, and in S1 and S2 Tables.

**Table 1:** Descriptive statistics.

	Group A				Group B				
	- FECD patients -				- Healthy subjects -				
	Mean	SD	Median	Range	Mean	SD	Median	Range	*P* value **
**Age (years)**	57.6	8.574	57.5	(42–74)	57.1	8.54	57.1	(41–74)	0.854
**VA (logMAR)**	0.25	0.376	0.1	(0–1.3)	0.015	0.048	0.0	(0–0,2)	0.01
ST mean reflectivity *	109.25	1.585	110	(106–112)	104.55	1.877	104.5	(102–108)	< 0.01
SN mean reflectivity *	109.05	1.791	110	(105–111)	105.60	1.903	106	(102–110)	< 0.01
IT mean reflectivity *	109.55	1.932	110	(103–111)	105.10	3.024	104.5	(102–111)	< 0.01
IN mean reflectivity *	109.45	1.959	110	(106–112)	104.55	2.114	104	(102–110)	< 0.01
**Endothelial CC (n. of cells)**	13.15	20.234	0.00	(0–59)	136.3	20.863	135	(105–182)	< 0.01
**ECD (cells/mm**^**2**^**)**	629.4	683.435	393.5	(0–1841)	2613.5	168.585	2643	(2207–2867)	< 0.01
**CV**	15.8	17.465	8.00	(0–46)	28.65	4.356	29	(20–37)	0.068
**Hex (%)**	25.1	26.999	16.5	(0–70)	65.75	2.531	65.5	(62–70)	< 0.01
**CCT (μm)**	564.45	70.3	557.5	(463–699)	544.95	24.377	546	(496–585)	0.301

VA: visual acuity; FECD: Fuchs’ endothelial corneal dystrophy; SD: standard deviation; ST: supero-temporal; SN: supero-nasal; IT: infero-temporal; IN: infero-nasal; CC: cells count; ECD: endothelial cell density; CV: Coefficient of Variation; Hex: Percentage of Hexagonal cell; CCT: central corneal thickness.

* = reflectivity (*R*) was quantified (*R* ∈ [0, 255]) by Image J 1.50 software.

** = If data were normally distributed (e.g. age), then the Two-sample *t*-test was used, otherwise the Mann—Whitney U test was used.

**Table 2:** Biomicroscopy, OCT pattern and reflectivity.

	Group A	Group B
	- FECD patients -	- Healthy subjects -
	Percentage (n.)	Percentage (n.)
**Sex**		
M	20% (16)	20% (16)
F	80% (4)	80% (4)
**Grading Score by Biomicroscopy**		
0	0% (0)	100% (20)
1	0% (0)	0% (0)
2	20% (4)	0% (0)
3	35% (7)	0% (0)
4	25% (5)	0% (0)
5	10% (2)	0% (0)
6	10% (2)	0% (0)
**OCT Pattern (all quadrants)**		
Homogeneous (1)	0% (0)	80% (16)
Dotty (2)	80% (16)	20% (4)
Marmoreal (3)	20% (4)	0%
**OCT Pattern (single quadrant)**		
**ST quadrant**		
Homogeneous	0% (0)	85% (17)
Dotty / Marmoreal	100% (20)	15% (3)
**SI quadrant**		
Homogeneous	0% (0)	90% (18)
Dotty / Marmoreal	100% (20)	10% (2)
**IT quadrant**		
Homogeneous	0% (0)	80% (16)
Dotty / Marmoreal	100% (20)	20% (4)
**IN quadrant**		
Homogeneous	0% (0)	90% (18)
Dotty / Marmoreal	100% (20)	10% (2)

ST: supero-temporal; SN: supero-nasal; IT: infero-nasal; IN: infero-nasal

Informative OCT images were easily obtained in all participants. In particular, as regards the clinical classification by biomicroscopy, the grade 0 was found in 0% of patients of Group A, and in 100% of cases of Group B; the grade 1 was detected in no study participant; the grades 2, 3, 4, 5 and 6 were observed only in Group A in 20%, 35%, 25%, 10% and 10% of patients, respectively.

Of note, OCT patterns showed the following distributions in the two groups: patterns 1 (Fig 1) and 2 (Fig 2) were observed respectively in 80% and 20% of subjects in Group B; conversely, only patterns 2 and 3 (Fig 3) were observed respectively in 80%, and 20% of patients in Group A.

According to an in-depth sub-analysis of OCT pattern and reflectivity (*R*) distributions in each quadrant of inner area of cornea, some interesting differences between healthy control subjects and patients were disclosed (Tables 1 and 2). Specifically, the OCT reflectivity (*R* ∈ [0, 255]), was measured for each quadrant (the inner plane of the cornea was divided into four finite regions, called quadrants, each bounded by two half-axes). In this way, the following results were obtained for OCT reflectivity in group B: 104.55 ± 1.877, 105.6 ± 1.903, 105.1 ± 3.024, 104.55 ± 2.114 in the ST, SN, IT and IN quadrant, respectively. In contrast, we found higher reflectivity values in all regions in group A: 109.25 ± 1.585, 109.05 ± 1.791, 109.55 ± 1.932, 109.45 ± 1.959 in the ST, SN, IT and IN quadrant, respectively.

Overall, correlation analysis disclosed a positive relationship between OCT reflectivity and clinical classification (grading score obtained by biomicroscopy), as well as an inverse relationship between OCT findings and endothelium cell count (CC), or hexagonal cells percentage (Hex), i.e. pleomorphism. As expected, there exists a moderate degree of dependence among the four quadrants of inner surface of the cornea, even if we found some independent relationships between individual quadrants and other variables (Tables 3 and 4). Each relationship between variables showed a strong correlation coefficient for each group.

**Table 3:** Correlation coefficients between variables (Kendall's tau_b test).

	OCT Reflectivity			
	ST quadrant	SN quadrant	IT quadrant	IN quadrant
	*Kendall's tau_b*			
**Age**	0.181	0.198	0.145	0.095
*(P value)*	0.134	0.104	0.232	0.432
**VA logMAR**	0.301^*^	0.266^*^	0.270^*^	0.257^*^
*(P value)*	0.020	0.042	0.039	0.048
**Biomicroscopy**	0.578^**^	0.482^**^	0.474^**^	0.519^**^
*(P value)*	0.000	0.000	0.000	0.000
**ST quadrant**	1.000	0.559^**^	0.625^**^	0.567^**^
*(P value)*	.	0.000	0.000	0.000
**SN quadrant**	0.559^**^	1.000	0.497^**^	0.591^**^
*(P value)*	0.000	.	0.000	0.000
**IT quadrant**	0.625^**^	0.497^**^	10.000	0.581^**^
*(P value)*	0.000	0.000	.	0.000
**IN quadrant**	0.567^**^	0.591^**^	0.581^**^	10.000
*(P value)*	0.000	0.000	0.000	.
**CC**	-0.570^**^	-0.360^**^	-0.414^**^	-0.507^**^
*(P value)*	0.000	0.003	0.001	0.000
**ECD**	-0.582^**^	-0.412^**^	-0.460^**^	-0.457^**^
*(P value)*	0.000	0.001	0.000	0.000
**Hex**	-0.399^**^	-0.418^**^	-0.277^*^	-0.392^**^
*(P value)*	0.001	0.000	0.020	0.001
**CCT**	-0.001	0.018	-0.084	-0.015
*(P value)*	0.991	0.878	0.471	0.897

ST: supero-temporal; SN: supero-nasal; IT: infero-nasal; IN: infero-nasal; VA: visual acuity; CC: cells count; ECD: endothelial cell density; CV: Coefficient of Variation; Hex: Percentage of Hexagonal cell;

**Correlation is significant at the 0.01 level (2-tailed).

*Correlation is significant at the 0.05 level (2-tailed).

**Table 4:** Correlation coefficients between variables (Spearman's rho test).

	OCT Reflectivity			
	ST quadrant	SN quadrant	IT quadrant	IN quadrant
*Spearman's rho*				
**Age**	0.238	0.266	0.191	0.139
*(P value)*	0.139	0.098	0.237	0.394
**VA logMAR**	0.389^*^	0.328^*^	0.337^*^	0.347^*^
*(P value)*	0.013	0.039	0.034	0.028
**Biomicroscopy**	0.751^**^	0.621^**^	0.578^**^	0.669^**^
*(P value)*	0.000	0.000	0.000	0.000
**ST quadrant**	10.000	0.701^**^	0.752^**^	0.715^**^
*(P value)*	.	0.000	0.000	0.000
**SN quadrant**	0.701^**^	10.000	0.623^**^	0.715^**^
*(P value)*	0.000	.	0.000	0.000
**IT quadrant**	0.752^**^	0.623^**^	10.000	0.734^**^
*(P value)*	0.000	0.000	.	0.000
**IN quadrant**	0.715^**^	0.715^**^	0.734^**^	10.000
*(P value)*	0.000	0.000	0.000	.
**CC**	-0.753^**^	-0.527^**^	-0.580^**^	-0.672^**^
*(P value)*	0.000	0.000	0.000	0.000
**ECD**	-0.760^**^	-0.577^**^	-0.611^**^	-0.648^**^
*(P value)*	0.000	0.000	0.000	0.000
**Hex**	-0.588^**^	-0.579^**^	-0.411^**^	-0.536^**^
*(P value)*	0.000	0.000	0.008	0.000
**CCT**	-0.009	0.006	-0.124	-0.045
*(P value)*	0.954	0.971	0.446	0.782

VA: visual acuity; ST: supero-temporal; SN: supero-nasal; IT: infero-nasal; IN: infero-nasal; CC: cells count; ECD: endothelial cell density; CV: Coefficient of Variation; Hex: Percentage of Hexagonal cell;

**Correlation is significant at the 0.01 level (2-tailed);

*Correlation is significant at the 0.05 level (2-tailed).

## Discussion

In the present study, the corneal endothelium features by means of 3D AS-OCT were evaluated in patients with FECD and in healthy subjects. The feasibility of AS-OCT in corneal dystrophy is widely recognized in the diagnostic field [12,18,19], and in selection or planning of different surgical procedures [20,21]. In this work, we focused on the potential, additional information provided by the 3D images obtained with AS-OCT. This device produces cross-sectional and en-face images of the cornea, thus resulting in a 3D cube of 128 horizontal scan lines, each composed of 512 A-scan. For our purposes, this cube was turned upside down by the in-built OCT software, and the reflectivity of the inner corneal layer was analyzed quantitatively and qualitatively.

As regards the OCT pattern analysis, the endothelium appearance showed three different models. The pattern 1 (or ‘homogeneous’), detected only in healthy subjects and in no affected eyes, was characterized by a uniform reflectivity of the endothelium, mainly appearing as blue (Fig 1). The mean and the maximum reflectivity of this pattern was relatively low if compared with the others (‘dotty’ or ‘marmoreal’).

The pattern 2 was found in all patients with mild or moderate FECD, and in only four eyes of the control group. It was characterized by an increased mean and maximum reflectivity and the presence of multiple hyper-reflective orange-yellowish points. For all these reasons, we defined this pattern as ‘dotty’ (Fig 2).

The pattern 3, detected only in four eyes with severe FECD associated with corneal edema, was defined as ‘marmoreal’ due to a mottled appearance of the corneal endothelium with a variable number of hyper-reflective regions (Fig 3).

We strongly believe that the presence of abnormal accumulation of macromolecules with irregularities in corneal water content and collagen fiber diameter may influence the light scattering of OCT signal trough the cornea, thus leading to the pattern 2 or 3. A similar mechanism has already been described by Chu et al in 2017 [22]. Indeed, the authors analyzed the backscattered light from the corneal tissue by a Pentacam Scheimpflug densitometry, stating that both abnormalities in corneal thickness and composition may contribute to change its optical properties in FECD patients.

As is known, the dying endothelial cells generally leave ‘empty’ spaces that are replaced by the expansion of adjacent cells, thus resulting in nodular formations [12]. Accordingly, the latter might explain the presence of hyper-reflective points in the FECD patients (typical in the ‘dotty’ pattern). However, these hyper-reflective points were also detected in 20% of healthy eyes, but unlike FECD eyes, were concentrated in one or maximum two quadrants. In these cases, we hypothesized that the OCT findings may be due to early focal defects in the corneal endothelium, which are undetectable either by biomicroscopy or specular microscopy.

In the advanced stages of the disease, there is generally an increased corneal thickness, more pronounced in the central cornea than in the periphery [10], and a corneal edema with epithelial defects, which imply an abnormal passage of the OCT signal through the corneal layers. This altered, non-homogeneous structure of the cornea, as in severe FECD, may explain the mottled appearance of the internal corneal surface observed specifically in pattern 3.

Since endothelial cells are in general mainly damaged in the central and paracentral zones [23,24], we firmly state that the 3D AS-OCT scanning protocol may represent an additional, suitable tool to detect or confirm the majority of alterations occurring in FECD patients at the different stages of disease.

Although some of our results should be interpreted as preliminary, in all study participants the age was positively related with the endothelial cells loss or other alterations detected by specular microscopy. These correlations were obviously stronger in the affected eyes probably due to the variation in cell size (polymegathism) and in cellular morphology (pleomorphism), which may prevent the diagnostic tool (i.e. the specular microscopy) from performing a regular sampling. Indeed, these results were typically observed in the advanced stages [25].

Interestingly, in all participants the age was also positively related to the reflectivity of the entire surface of the inner cornea (i.e. the four quadrants). Particularly, older patients showed an increased reflectivity in the supero-temporal area.

In group A, patients with a severe clinical grading score showed an increased mean reflectivity both in supero-temporal and infero-temporal quadrants. On the contrary, the reflectivity in all quadrants was inversely correlated to the endothelial cells count, thus confirming our hypothesis that healthy subjects with a normal CV and ECD may show a low reflectivity if compared with patients with severe FECD.

On the other hand, the results of our study suggest that confluent guttae associated with moderate stages of disease were more likely to appear as hyper-reflective points, if compared with non-confluent guttae of early stages. Moreover, in case of severe stages characterized by confluent corneal guttae > 5mm, 3D AS-OCT imaging showed a mottled appearance of the inner corneal surface, suggesting the presence of larger areas of endothelial defects or DM abnormalities.

Although FECD is considered a relatively rare pathology, a limit of the current study is represented by the relative small size of patients included. In future, our results should be confirmed and validated by a larger number of FECD patients and compared with different OCT technologies.

In sum, it can be stated that 3D AS-OCT endothelium reflectivity has an inverse relationship with the corneal endothelium integrity. Therefore, patients with a normal endothelium have a greater probability to show a homogeneous pattern of reflectivity, compared with patients with moderate or severe FECD. Furthermore, 3D AS-OCT is a non-invasive imaging modality that provides an in-depth visualization of the inner surface of the cornea. Since a very early detection of corneal alterations may be of great importance in the next future regarding a possible medical therapy, our findings suggest that OCT is a useful tool in investigation of fine, structural abnormalities of the inner corneal layers, thus potentially representing a valuable support in the setting of FECD diagnosis.

## Supporting information

S1 Table Data set of patients with Fuchs’ Endothelial Corneal Dystrophy (CASES). (XLSX)

S2 Table Data set of healthy participants (controls). (XLSX)
